# Supplementary material for: Genome-wide CRISPR Screens in T Helper Cells Reveal Pervasive Crosstalk between Activation and Differentiation
Source: Cell. 2019 Feb 7;176(4):882–896.e18. doi: 10.1016/j.cell.2018.11.044 (PMC6370901; doi:10.1016/j.cell.2018.11.044)
Supplement: Data S2. Processed Data from All the Steps of the Analysis, Related to Figure 1 [file mmc2.zip › supplemental data/motif analysis/Bhlhe40_homer/homerResults/motif7.similar.html]

motif7

## Information for motif7

T
C
A
G
A
G
T
C
A
G
T
C
C
G
T
A
C
G
T
A
A
G
C
T
T
G
A
C
T
C
A
G
A
C
T
G
G
T
C
A
  
Reverse Opposite:  

C
A
G
T
G
T
A
C
A
G
T
C
A
C
T
G
C
T
G
A
C
G
A
T
A
C
G
T
A
C
T
G
A
C
T
G
A
G
T
C
  

|  |  |
| --- | --- |
| p-value: | 1e-13 |
| log p-value: | -3.214e+01 |
| Information Content per bp: | 1.738 |
| Number of Target Sequences with motif | 55.0 |
| Percentage of Target Sequences with motif | 4.58% |
| Number of Background Sequences with motif | 567.1 |
| Percentage of Background Sequences with motif | 1.33% |
| Average Position of motif in Targets | 95.1 +/- 54.8bp |
| Average Position of motif in Background | 83.9 +/- 61.4bp |
| Strand Bias (log2 ratio + to - strand density) | -0.0 |
| Multiplicity (# of sites on avg that occur together) | 1.09 |
| Motif File: | file (matrix) reverse opposite |

### Similar de novo motifs found

|  |  |  |  |  |  |  |  |
| --- | --- | --- | --- | --- | --- | --- | --- |
| Rank | Match Score | Redundant Motif | P-value | log P-value | % of Targets | % of Background | Motif file |
| 1 | 0.867 | A C T G C T G A C T A G A G T C A G T C C G T A C G T A A C G T T A C G A C T G C T A G C G T A | 1e-13 | -30.470213 | 1.67% | 0.17% | motif file (matrix) |
| 2 | 0.938 | A G T C A G T C A C T G C G T A C G A T A C G T C T A G A T C G | 1e-12 | -29.533909 | 9.33% | 4.38% | motif file (matrix) |
